# Small and medium-sized enterprises failure in providing workers’ rights concerning Sustainable Development Goals-2030 in Pakistan

**DOI:** 10.3389/fpsyg.2022.927707

**Published:** 2022-10-06

**Authors:** Rana Tahir Naveed, Mahmood Rehmani, Munnawar Naz Khokhar, Syed Raza Ullah Shah, Anis Ali, Sadaf Shahzadi, Huda Irshad

**Affiliations:** ^1^Department of Economics and Business Administration, University of Education, Lahore, Pakistan; ^2^Department of Management Sciences and Economics, Grand Asian University Sialkot, Sialkot, Pakistan; ^3^Department of Management Sciences, COMSATS University Islamabad, Islamabad, Pakistan; ^4^Department of Business Administration, University of Sialkot, Sialkot, Pakistan; ^5^Department of Management, College of Business Administration, Prince Sattam Bin Abdulaziz University, Al-Kharj, Saudi Arabia

**Keywords:** SMEs, labor rights, industries, sustainability, Sustainable Development Goals

## Abstract

Small and medium-sized enterprises (SMEs) play an enormously crucial part in the modern world economy, demonstrating the most unique and incredible ground-breaking system. SMEs’ employment statistics and future worker needs make it a focus of policies among rising economies, and Pakistan is no exception. The working conditions in SMEs diverge from industry to industry; however, irrespective of the industrial categorization, the SMEs are failing to protect the workers’ rights in the perspective of Sustainable Development Goals (SDGs). The interpretivism paradigm and purposive sampling, narrative inquiry, and analysis method have been adopted to gain in-depth knowledge of workers’ rights in SMEs concerning the SDGs. Results revealed that Pakistan-based SMEs argue to be financially weak and perceived as less equipped to adhere to the world’s standards. Highlighted issues in failure to adhere to worker’s rights include lack of financial resources, top management’s commitment, regulatory framework, SDG awareness, strategic planning, and the dire need for expert guidance and consultation in translating goals to work environments.

## Introduction

Small and medium-sized enterprises (SMEs) are a significant engine of financial expansion and socioeconomic advancement (Taylor and Murphy, 2004; Najeeb, 2021). SMEs include all private sector small and medium-sized industries, businesses and economic entities (Durst and Runar Edvardsson, 2012). SMEs have a vital role in implementing the plan set under Sustainable Development Goals (SDGs) (Verboven and Vanherck, 2016) as this sector creates jobs that diminish unemployment and poverty at a lower level. The SMEs have generated 90% of new positions in the formal sector (Razak et al., 2018; Joan et al., 2022). Accordingly, the World Bank report has also pointed out that in the future, 600 million jobs will be required to take up the emergent worldwide workforce needs, mainly in Asia and sub-Saharan African regions, where annual growth rates may exceed 2.7% (World Bank, 2019).

The SMEs are captivating a prominence in fulfilling the business-linked SDGs for sustainable economic growth for decent work and generating a constructive shove for an elevated worth of living, education, and health for all. These enterprises significantly contribute to poverty reduction, particularly in the rural areas for uneducated but skilled men and women. They support low-income families to run their expenditures by earning at a small level (World Bank, 2019). The SDGs demand converting plans with systemic modification in financial markets and institutions work (World Bank, 2019).

In Pakistan, there were 5.2 million SMEs reported last year in the press (Najeeb, 2021). These SMEs make up nearly 90% of exclusive private businesses. These enterprises contain manufacturing units, service suppliers, and startups working at different stages. The SMEs use 78% of the non-agricultural workforce, accounting for 25% of manufacturing exports and 30% of Pakistan’s GDP (Shah and Syed, 2018). The Small and Medium Enterprises Development Authority (SMEDA) was inaugurated in 1998 for SMEs support and smooth progress. It is not just an SME policy-advisory institute for the government, excluding facilitating the vision of other stakeholders in dealing with their SME growth schema. The SMEs in Pakistan are low-income enterprises with fewer employees with less attention to SDGs, and only a few have adopted some SDGs-related agenda items (Subhan et al., 2013). In the absence of these agenda points’ implementation, the enterprises face numerous issues. If these enterprises adopt and implement these agenda items in the workplace, it would undoubtedly support their growth and workers in the workplace with ultimate benefits to the owners and further contribute to the national economy in Pakistan. Shah and Syed (2018) reported that SMEs do approximately 30% of the export in Pakistan, contributing 40% share of the total GDP of Pakistan. The SMEs also positively impact people’s lives as this sector provides them economic opportunities for earning and fulfilling their financial needs.

Pakistan has been a signatory of SDG 2030 from its beginning in 2015 (Qazi, 2021). The National Assembly of Pakistan adopted these goals in February 2016 and became the first country who adopts SDGs in 2030. Pakistan’s 12th 5 years plan and provincial level strategies were developed aligned with these SDGs. Seven SDG support units were established at the federal and provincial levels to enhance collaboration among the different government departments and non-government stakeholders to progress toward SDG 2030 (Qazi, 2021). This research aims to understand why SMEs management in Pakistan cannot fulfill the workers’ rights required by SDG 2030. Are they willing to contribute their role toward the achievement of SDG 2030? Is the government supporting SMEs to provide workers’ rights? It explored how SDGs developed on the international level are implemented in SMEs in Pakistan. Primarily, there is very less research seeking the applicability of SDGs on the SMEs for the worker’s rights, and hence, this article will contribute remarkably to the existing knowledge.

### Literature review

Zou et al. (2021) explored the SMEs to execute corporate social responsibility (CSR) for an economic rise to get deep acquaintance with various CSR blockades. Their qualitative thematic analysis of Sialkot-based SMEs produced five interrelated themes of CSR barricades: lack of resources and policies required top management’s pledge, need for CSR information, and inactive buyer behavior (Islam et al., 2021; Rehmani et al., 2022a,b). Zou et al. (2021) analyzed that resource limitations are the key barrier preventing SMEs from being affianced by CSR actions. Through responsiveness to obstructions, policymakers and experts can take the required measures to develop CSR applications in SMEs. Organizations can gain a competitive edge in emerging markets, so focusing on CSR and corporate social performance is necessary (Crişan-Mitra et al., 2020). Lotfi et al. (2021) and Hamid et al. (2021) both revealed that many supply chains have the assurance to avoid the abuse of workers’ rights, an essential part of social sustainability in their distant supply chains. They have provided a way to recognize how supply chains do not defeat the infringement of labor’s rights by adopting the UN suggested SDGs on the social basis of the “doughnut model” regarding labor rights in supply chains. They have developed the “sustainable supply chain doughnut model” regarding the SDGs, through which they have investigated the breach of labor rights. A study by Lǎzǎroiu et al. (2020a) focused on green public procurement driving the economy as an environment policy mechanism for sustainable goods and services.

A similar study by Ullah et al. (2021) gave an understanding of how government enticement (economic and non-economic) manipulates green modernism and Sustainable Development Goals (SDGs) association in SMEs. The research suggested that SMEs focus on implementing green modernization and technology to keep the surroundings and ease society. The results of this empirical study of 204 Pakistani SMEs pointed out that green concepts have an essential sway on SDGs, public growth, and environmental behaviors. Furthermore, government support significantly strengthens the relationship between green innovation and environmental practices, while it does not moderate the path between green innovation and community development. Moreover, the government must financially support the SMEs, so that SMEs may facilitate the labor and workers by implementing the SDGs. Corporate governance systems of organizations shape operational environmental sustainability (Lǎzǎroiu et al., 2020b; Rehmani et al., 2020).

In this study, we explore the failure of SMEs Management to fill the worker’s rights as required by SDG 2030. According to the International Labor Organization (ILO) conventions illustrated, workers’ rights research defines it by using the terms “freedom of association, collective bargaining, the abolition of forced or compulsory labor, equality of opportunity and treatment in employment and occupation, protection of children and young persons, wages, working hours, safety, and the legal employment relationship.” We have studied SMEs’ workers’ rights in Pakistan following the SDGs agenda 2030. We explore among the 17 goals directly related to workers’ rights, and SMEs can contribute to it. We studied why SME management has failed to fulfill the workers’ rights required by these related SDGs. How these workers’ rights can be secured; to what extent the government may support SMEs; and how can these SMEs support Pakistan’s national economic growth?

The SMEs are enterprises with low budgets that contribute to the country’s economy. There appears to be no universally accepted definition of SMEs. The SMEs definitions are still confusing concepts as they vary. For example, SMEs have less than 500 workers in France. The description may vary according to the kind of business in Japan, where mining, manufacturing, shipping, and building industries are defined as SMEs with less than 300 workers. In Ghana, SMEs are defined as an enterprise with an annual income of “23,700–2,370,000” dollars (Gibson and Van der Vaart, 2008). In Thailand, gross national income (GNI) per capita is five times higher than Ghana; SMEs are considered companies that generate income from 84,400 to 8,440,000 dollars. Tables 1, 2 shows the definitions described by different countries, such as, Germany, Belgium, United Nations and Canada. This helps understand the criteria used by various institutions globally.

**TABLE 1:** Small and medium-sized enterprise (SME) definitions used by multilateral institutions.

Institutions	Maximum Number of Employees	Maximum Revenue or Turn over (s)	Maximum Assets (s)
**World Bank**	300	15,000,000	15,000,000
**MIF-IADB**	100	3,000,000	(None)
**African Development Bank**	50	(None)	(None)
**Asian Development Bank**	No Official Definition	Uses only definitions of individual	National Governments
**UNDP**	200	(None)	(None)

Source: Gibson and Van der Vaart (2008).

**TABLE 2:** The worldwide description of SMEs.

Countries	Definition	Measurements
Germany	Employees not exceeding 225, annual turnover not exceeding 50 million Euro	Employment and assets
Belgium	100 Employees, annual turnover of not then 50 million Euro	Employment
United Nations	1,500 Employees and $50 million Euro	Employment and assets
Canada	Not more than 500 Employees for manufacturing and 50 employees for service Industries	Employment

Source: Adapted from Cunningham and Rowley (2008, 355–356).

### Small and medium-sized enterprises in Pakistani milieu

No standardized description of SMEs exists in Pakistan (Dar et al., 2017). The “SME Bank, SMEDA, Pakistan Bureau of Statistics (PBS), and State Bank of Pakistan (SBP) all have defined SMEs differently. The PBS accepts an SME based on employee enrollment, while the SBP’s description of the SME is the nature of the business and the number of employees. The SME bank has elaborated many assets as the standard. In schedule 05 to Companies Ordinance 2015, the Security and Exchange Commission of Pakistan (SECP) mentioned large, medium, and small enterprises where SMEDA defined SME on the basis of employee strength and productive resources.

### Small and medium-sized enterprises in Pakistan

Small and medium-sized enterprises (SMEs) play a significant role in developing a country’s economic, industrial, and social domains. Rohra and Panhwar (2009) mentioned that most developed states grant SMEs’ significance in supporting their economy. SMEs have a vital role in the growth as it has been a source of job creation and revenue. Pakistan-based SMEs play a critical role in economic development and technological innovation. SMEs are playing a part in poverty reduction and increasing the state economy. In Pakistan’s financial system, these enterprises are uplifting employment. Like other developing nations, the state economy directly impacts the SME sector (Khalique et al., 2011). According to the Economic Census of Pakistan 2020, the GDP growth is 3.94% against the visualized target of 2.1%, transmittals have made remarkable records, and exports are touching 25 billion dollars in the extroverted financial year. Till June 2021, Pakistan’s finance was 437.57 billion rupees which were 6.57% of the total private sector income, where 172,893 SMEs were the borrower. Table 3 shows the definition of SMEs in Pakistan and also elaborates the criterion and scale differences.

**TABLE 3:** Small and medium-sized enterprises definition in Pakistan.

Institutions in Pakistan	Criterion	Medium scale	Small scale
Small and medium enterprise development authority (SMEDA)	No. of employees (<250 employees Productive Asset	Between 36 and 99	Between 10 and 35
SME Bank Federal Bureau of Statistics State bank of Pakistan	Productive Total Assets Federal Bureau of Statistics Nature of Business (Manufacturing Trade/Services) No. of employees Capital employed Net sale value	Assets 20–40 million PKR Over 100 million PKR Less than 250 employees and less than 100 million PKR assets for manufacturing. Less than 50 employees and less than50 million PKR for trade/services. Net sales less than 300 million PKR	2–20 million PKR Less than 100 million No. of Employees N/A Less than 10 employees Less than 250 employees and less than 100 million PKR assets for manufacturing. Less than 50 employees and less than 50 million PKR for trade/services. Net sales less than 300 million PKR
Securities and Exchange Commission of Pakistan	Company which has annual gross revenue (grants, income. Subsidies, donations) including Other income/revenue less than Rs. 200 million.	A non-listed company which is not a : (a) Public Interest Company; or (b) Large sized Company or (c) Small Sized Company other than a non-listed public company	Other than a non-listed public company: (a) Paid up capital not exceeding Rs. 25 million, and (b) Turnover not exceeding Rs.100 million.

Source: Dasanayaka (2008, 71); SMEDA (2011).

### Exploring worker’s rights in small and medium-sized enterprises concerning Sustainable Development Goals

For this study, we have divided SDG into three groups. In Group 1, all the goals directly related to workers’ rights in SMEs are included. Group 2 consists of the SDGs that are related to the environment. In Group 3, only SDGs associated with the ideal layer are included in Group 1.

#### Group 1

SDG 1: Poverty reduction in all its forms and domains.

SDG 2: Hunger reduction, food security, and promote sustainable agriculture.

SDG 3: Guarantee health services and endorse wellbeing plans for all age groups.

SDG 4: Ensure a wide-ranging and reasonable value-added education program and uphold lifetime opportunity for all with no discrimination.

SDG 5: Gender equality and women and girls empowerment.

SDG 8: Promote sustained, broader and sustainable economic growth and productive employment and decent workplace.

SDG 10: Condense inequalities between inter and intra nations.

SDG 16: Promote peaceful and inclusive societies for sustainable development, provide access for all and build effective, accountable, and inclusive institutions at all levels.

SDG 17: Strengthen the means of implementation and revitalize the global partnership for sustainable development.

#### Group 2

SDG 6: Ensure availability and sustainable management of water and sanitation for all.

SDG 7: Ensure access to affordable, reliable, sustainable and modern energy to all.

SDG 9: Build resilient infrastructure, promote inclusive and sustainable industrialization, and foster innovation.

SDG 13: Take urgent actions to combat climate change and its impact.

SDG 14: Conserve and sustainably use the oceans, seas, and marine resources for sustainable development.

SDG 15: Protect, restore, and promote sustainable use of terrestrial ecosystems, sustainably managed forests, combat desertification, halt and reverse land degradation, and halt biodiversity loss.

#### Group 3

SDG 11: Sustainable city and community.

SDG 12: Insurance of sustainable consumption and production patterns.

Our primary focus in this study is the goals given in Group 1. The workers’ rights studied concerning the objectives given in Group 1 include basic needs, health, education, income and work, peace and justice, social parity, gender egalitarianism, and networks.

## Methodology

Following the interpretivism paradigm, this research used a narrative analysis approach. As the study explores the SDGs in 2030, the employees’ perspective is chosen for analysis. It is essential to understand the individual and his perception as everyone sees the world their way. This method provides a greater understanding of the human condition and perceptions. The concept of interpretivism sheds light on the human nature that reality is viewed and interpreted according to the brain’s conditioning, which comes from experience. It provides a perspective of the situation of SGDs in the future in the interviewees’ minds and shares their understanding. A qualitative approach has been adopted to gain in-depth knowledge of SME workers’ rights concerning the SDGs described in Group 1. The purposive sampling technique is used to select the respondents, as Abbas and Hafeez (2021). We contacted the management of SMEs from surgical, leather, and sports goods to ask why the failure to fulfill the workers’ rights in the Context of SDG 2030. These respondents were CEOs, GMs, and managers. The researchers know these professionals personally. The data were collected through interviews conducted *via* phone and physically. After collecting data, narrative analysis was used to analyze it (Abbas and Hafeez, 2021). The sample size is limited as few interviews of well-known acquaintances provided the necessary details to conduct the narrative analysis. Collecting and analyzing the information shared by people help describe different experiences and offer their interpretations. Similarly, in narrative research interviews, information from interviews is written and compared with other SDG research, and then, a link is determined to explain the findings better.

### Analysis and findings

The narrative analysis of this study showed that there are different reasons why SMEs cannot provide the workers and rights as required by SDG 2030. These reasons can be categorized into five groups based on the interviews conducted with the management of different SMEs. These categories are lack of finance, lack of top management commitment, lack of regulatory framework, lack of SDGs knowledge, and lack of strategic planning for SDGs.

#### 1. Lack of finance

Most of the respondents shared that lack of financial resources is the primary reason for not providing the workers’ rights as required by SDG 2030. The majority of interviewees shared that they are willing to provide all the rights to their workers as required by SDGs, but due to the limited financial resources, they are unable to fulfill this requirement:

*“Being an export-oriented SME, we have different challenges. Our operations are order-based, and price competition is very high, so we find it difficult to provide all the rights to the workers. Providing each facility involves cost and requires funds, so due to the availability of limited funds, we have to remain cost-effective.”* SME 1

*“We know that our workforce is the backbone of our business, and Sialkot has a unique type of workmanship. All our workers are earning enough to meet their personal and family needs. We have the piece-rate workers, which means more work we have more they do and make more. However, a small organization can’t afford to fulfill all their rights due to the non-availability of funds as our profit margins are meager.”* SME 7

*“It seems very hard to provide all the workers’ rights required by the International Standards. Registration with Social Security, EOBI, paying them for annual, sick, casual and maternity leaves, paying the overtime on premium rates and providing them health and safety facilities all need money beyond the affordability of a small organization.”* SME 7

The respondents expressed that if they have enough financial resources, they will provide all the rights to their workers and try to perform their role to achieve the SGDs 2030. They believe that they will be more productive if the workers are facilitated. Although the SMEs enable their workers, they can do more for their workers if they have funds.

#### 2. Lack of top management commitment

Another reason for failure in providing the workers’ rights as required by SGDs 2030 is a lack of top management commitment (Rehmani and Khokhar, 2018), as pointed out by the participants during the interview. They said:

*“The lack of top management commitment in providing workers’ rights as required by the GSDs is pronounced. For them, it is on least priority. Top management focused on business expansion, building expansion, and purchasing new lands. They are not interested in solving the issues workers face in their lives. They are not ready to give workers a permanent job and prefer to hire them on piece rate bases*.” SME 2

*“The top management is unwilling to take any step to improve workers’ lives. Even the working environment in the organization is not a decent working place and has many health and safety risks, and the top management does not have any plan to improve it. They are not willing to eliminate the discrimination especially based on gender. They are not willing to participate in any collaborative program to enhance the capacity building of their workers through training.”* SME 5

“*As we have some International certifications and our international brand is very conscious about the workers’ rights, our top management provides all the necessary resources for maintaining these certifications. However, there are primarily many issues related to worker rights, which have never been a priority by top management. Many things are good, but these are in the paper.*” SME 8

The respondents believe that the lack of top management commitment toward the workers’ rights according to SDGs is one reason for the failure to provide these rights. Although they are trying to provide some basic facilities to the workers, it requires more attention and support from the top management to fulfill their rights according to SDGs.

#### 3. Lack of enforcement

The interviewees also narrated that due to poor implementation of the laws related to the workers and the ineffectiveness of the government regulatory bodies responsible for protecting the workers’ rights, the workers are facing many difficulties:

“*Although labor laws are available and the government has announced the labor policy to protect the workers’ rights, which are very comprehensive and admirable. However, the implementation of these rules and regulations is fragile. Most workers do not have job security; they do not have a job contract from the employer and can be fired without fulfilling legal requirements.*” SME 9

“*Health and safety conditions in factories are worse, cleanliness, First aid arrangements. Fire fighting arrangements and emergency exit paths are not properly maintained. Portable water, sanitation facilities, and food serving arrangements are not per legal requirements. Although there are many departments the government has established for monitoring the availability of these facilities according to law, it seems that this monitoring is not effective*.” SME 13

The government should improve its mechanism to implement the regulations already developed to protect workers’ rights. Some effective channels of monitoring and motivation should be formed. According to the SDS, organizations that provide good facilities to their workers should be given some incentives and rewards.

#### 4. Lack of Sustainable Development Goals knowledge

The participants admitted that awareness of the SDGs is rare in Sialkot. On the subject, they have not heard or learned much about this. People have a scarce understanding of the SDGs 2030 and how they can contribute their roles to the achievement of these goals:

*“We are working on the different International Standard related to the environment, quality, health and safety, and social accountability. We have participated in different training and seminars related to these standards; however, any seminar or training was rarely held on SDGs 2030.”* SME 12

“*The SMEs do not sufficiently understand the details about the 17 SDGs in Sialkot, which are related to workers’ rights and the details about these are mostly unknown.”* SME 4

It was narrated that although the regulations related to labor rights are available, their implementation is not adequate. It is difficult for the workers to get their rights to the existing framework. The role of the government in monitoring regulator authorities related to workers’ rights should be practical and supportive for implementing existing regulations. According to the SDS, organizations that provide good facilities to their workers should be given some incentives and rewards.

#### 5. Lack of strategic planning for Sustainable Development Goals related to workers

The participants described that their business plans do not include any separate strategic planning for SDGs about workers’ rights. Being small-sized organizations, we are mainly occupied with our day-to-day operations:

*“SDGs related Planning is related to the Government or the big Organizations with a big workforce and enough resources. In small organizations, we don’t think that we can contribute to these SDGs, so we do not have a strategic plan for SDGs.”* SME 12

*“Workers are our main strength and we try our best to facilitate as much as we can; however, having some sort of separate strategic planning for SDGs related to workers’ rights is not possible.*” SME 15

In SMEs, there are no well-described business plans. They rarely have the strategic planning for the SDGs related to workers. Due to limited knowledge about the SDGs, they do not prepare any strategic goals related to these SDGs.

#### 6. Sustainable Development Goals related to workers’ rights implementation complexity

The interviewed persons expressed that they are competent in their core operations, and it is not easy for them to understand the SDGs and how to implement these in their current business setup:

*“For implementing such International requirements like SDGs, we need some experts to help us. Normally we hire some consultant; however, it is difficult to understand for our existing workforce and management staff, and we need a budget to pay for the consultant.”* SME 13

*“SDGs related to the workers demand a lot beyond our capacity. It is not easy that we can understand them and implement these requirements with any external support. It is complex and requires a lot of effort.”* SME 15

The respondent narrated the complexity of the implementation of SDGs related to workers’ rights. It is required to hire some professional services to help them perform in their organization. Also, it is necessary to enhance the understanding of their existing staff toward these SDGs.

## Conclusion

Small and medium-sized enterprises contribute significantly to private sector entities in developed and developing economies. The vital role of SMEs for extensive social-economic growth like employment chances gives them precedence for attaining the SDGs. These goals are sweeping arrangements calling for the functioning of all economies to perk up the populace everywhere. SDGs aim to reduce poverty, decrease inequality, and deal with climate change. SMEs in Pakistan work on a low budget and hardly afford sustainable development agenda in their workplace for laborers. Still, despite size and income, all shareholders, employees, and customers expect a similar pledge to sustainability from all small and medium businesses (Iftikhar et al., 2021). Being small can be an advantage, it usually means being additional supple and able to make arrangements quickly. In Pakistan, there are some limitations of financial assistance, low budget and income, lack of top management commitment, regulatory framework, less or no SDG knowledge, lack of strategic planning for workers, and SDGs related to workers’ rights implementation complexity (Zaman et al., 2022). SMEs management can be improved by adopting SDGs for employee betterment. It will provide auxiliary lead to a better working environment and job satisfaction to workers and enhance SMEs productivity.

### Recommendations

Based on the research and interviews conducted by the SMEs or top management owners, some gaps in the policymaking and implementation process must be addressed as a top priority. A few policy recommendations for the adoption of SDGs in SMEs are given as follows:

• The SMEs must adopt a long-term strategy for the implementation of SDGs. If SMEs adopt these goals, they will get an extensive customer range.
• The SMEs owners must follow their sustainability act through sustainability exposure such as an annual report, sustainability reports, and SDGs as a stencil for reporting.
• Government must encourage SMEs and facilitate them in adopting SDGs through some subsidies offers or financial assistance.
• The training programs for owners of SMEs must start nationally and internationally. This will encourage them and make them aware of the significance of SDGs adoption in their enterprises.
• The employees must also offer training and workshop for knowledge about these sustainability parameters.

### Limitations and scope

The data collected for this study was through interviews due to limited sample size. Few interviews were conducted simultaneously, i.e., cross-sectional, so the future research should expand to other cities of Pakistan with greater sample size and choose longitudinal research design to increase the generalizability of results for other countries. Purposive sampling has been used to fulfill the study’s need to seek the narrative of respondents, so the sample chosen was based on their knowledge of SDGs. Future researchers should use a different sampling technique, such as cluster sampling, to get a more significant portion of the population to offer their insight on the matter.

A conceptual model can be incorporated in the future for sustainable development as the awareness of SGDs in developing countries is low. The model may include SME performance, SDGs awareness, and financial assistance. Other factors can be added depending on the economic conditions of the country. Through research, the knowledge gap can be overcome and create awareness among workers. When the SMEs are self-sustainable, the poverty levels of society can be reduced, and necessities can be accessed more easily than before. With passing the time, natural resources are depleting, due to which SMEs need to be self-sustained. To do so, the UN has provided a future guideline for SDGs compliance to manage the organization in these challenging times. Managers can learn from this study and accordingly meet the requirements of SDGs to make sustainable decisions.

## Data availability statement

The original contributions presented in this study are included in the article/supplementary material, further inquiries can be directed to the corresponding author.

## Author contributions

RN proofread the first draft composed by MR. MK made some improvements to the draft. SRS and SS helped with the data collection. AA refined the statistical analysis. HI improved the draft to be ready for publication. All authors contributed to the article and approved the submitted version.
